# Pretraining of Embodied Recurrent Networks Bridges the Gap Between Artificial and Cortical Neural Activities

**DOI:** 10.3390/biomimetics11080569

**Published:** 2026-08-09

**Authors:** Xiangdong Bu, Hongru Jiang, Tianruo Guo, Heng Li, Yao Chen

**Affiliations:** 1 School of Biomedical Engineering, Shanghai Jiao Tong University, Shanghai 200240, China; bxd0324@sjtu.edu.cn (X.B.); hongrujiang@sjtu.edu.cn (H.J.); 2 Graduate School of Biomedical Engineering, University of New South Wales, Sydney, NSW 2052, Australia; t.guo@unsw.edu.au; 3 Department of Physical Education, Shanghai Jiao Tong University, Shanghai 200240, China

**Keywords:** motor control, motor primitive, recurrent neural network, musculoskeletal arm model, neural population dynamics

## Abstract

Task-driven recurrent neural networks (RNNs) have been widely employed as tools for investigating neural dynamics in neural motor control research by modeling the motor cortex. RNNs are often implicitly assumed to learn the underlying computational mechanisms in accordance with biological neural circuits. However, the brain network has a highly structured and specific network connectivity and during individual development the motor cortex has acquired a rich repertoire of behavioral primitives via continuous learning of body control. Considering that the task-driven RNNs are often initialized randomly and trained directly on the specific task, how much these models can truly reveal about the motor cortex is still a crucial question awaiting further research. In this study, we propose a method for modeling the motor cortex pretrained on single reaching skills. Specifically, we use an RNN, receiving sensory feedback and task inputs, as the controller to produce motor commands that drive a musculoskeletal arm model. This model can perform reaching movements along a mini-jerk trajectory between arbitrary points in the workspace, prior to training on specific tasks. The model pretrained on single-reach task has more similarity with real neural data both on a neural geometry and neural dynamics level in center-out (CO) and random target touch (RTT) tasks than models directly trained on these tasks. Surprisingly, we observed the opposite pattern in a double-reach (DR) task, in which two targets appeared simultaneously, rather than presenting the next target after the completion of the prior movement as in the RTT task. This suggests that sequential movements are planned as an integrated unit, and this capability may be implemented at the level of motor cortical circuits. In summary, our results suggest that endowing the network with capabilities beyond the immediate task demands—through more systematic training or other methods—can help better understand the dynamics of biological neural circuits.

## 1. Introduction

As one of the most essential functions for animals, motor control has long been a key focus of neuroscience. Traditionally, research on neural motor control primarily involved training animal subjects to perform specific motor tasks while recording neuronal activity in the motor cortex via implanted electrodes [1,2,3,4,5,6]. In recent years, advancements in large-scale electrophysiological recording technologies have shifted attention from single-neuron activity to coordinated population-level dynamics [7,8,9,10,11], establishing the dynamical systems perspective as a predominant framework for interpreting motor cortical activity [12,13,14,15,16]. Recurrent neural networks (RNNs), as inherently dynamical systems, have become powerful modeling tools in this context [17,18], yielding insights into context-dependent computation [19], muscle activity generation [20], motor primitives [21], and motor preparation [22].

Approaches to modeling biological neural networks with RNNs can be broadly conceptualized into two major frameworks. Data-driven paradigms [23,24] optimize network models to directly fit empirically recorded neural activities, achieving high reconstructive fidelity but remaining inherently correlational: they describe what the neural dynamics look like without revealing why particular dynamical motifs emerge or how they are shaped by behavioral demands. Task-driven paradigms [20,25], currently the more prevalent methodology, train RNN connectivity weights using task conditions from animal experiments as inputs, with behavioral kinematics or targets as outputs. The trained model is then compared with recorded neural data at single-neuron or population levels [26,27] to infer the computational mechanisms by which biological subjects perform the same task [28,29,30].

A central assumption of the task-driven approach is that if a network solves the same task as an animal and recapitulates its neural activity patterns, then the two may employ similar underlying computational mechanisms. However, recent findings of substantial solution degeneracy—networks trained on identical tasks from different random initializations converge to qualitatively different dynamical solutions, only a subset of which align with neural data [31]—have challenged this assumption. This degeneracy arises precisely because randomly initialized networks lack the structured priors that biological circuits acquire through evolution and development. In contrast, the brain is pre-initialized at birth with highly optimized connectivity [32], and through millions of years of evolution and years of sensorimotor experience during development, the motor cortex amasses a rich, versatile repertoire of behavioral skills and motor primitives. Consequently, when naïve experimental subjects confront a novel laboratory paradigm, they already harbor foundational motor capabilities, and their learning process is fundamentally characterized by leveraging pre-existing skills to explore task rules.

This insight converges with a growing body of work on pretraining in computational neuroscience. Compositional pretraining—training on constituent task primitives before the full task—improves computational efficiency and matches animal behavior on complex cognitive tasks [33]. Unsupervised pretraining in mouse visual cortex produces representations that accelerate downstream learning, providing direct biological evidence for pretraining-like mechanisms in the brain [34]. Warm-up training with random noise improves uncertainty calibration in neural networks, paralleling the role of spontaneous activity in developing circuits [35], while biologically structured initialization mimicking preconfigured connectivity significantly improves RNN-cortical alignment [32]. These studies suggest that how a network is initialized and pretrained may be as important as its architecture in determining its computational properties. However, to our knowledge, no prior study has systematically investigated whether pretraining on a broad repertoire of embodied motor skills—rather than on individual cognitive tasks or through random noise exposure—can bridge the gap between artificial RNN dynamics and motor cortical activity.

**Figure 1:**
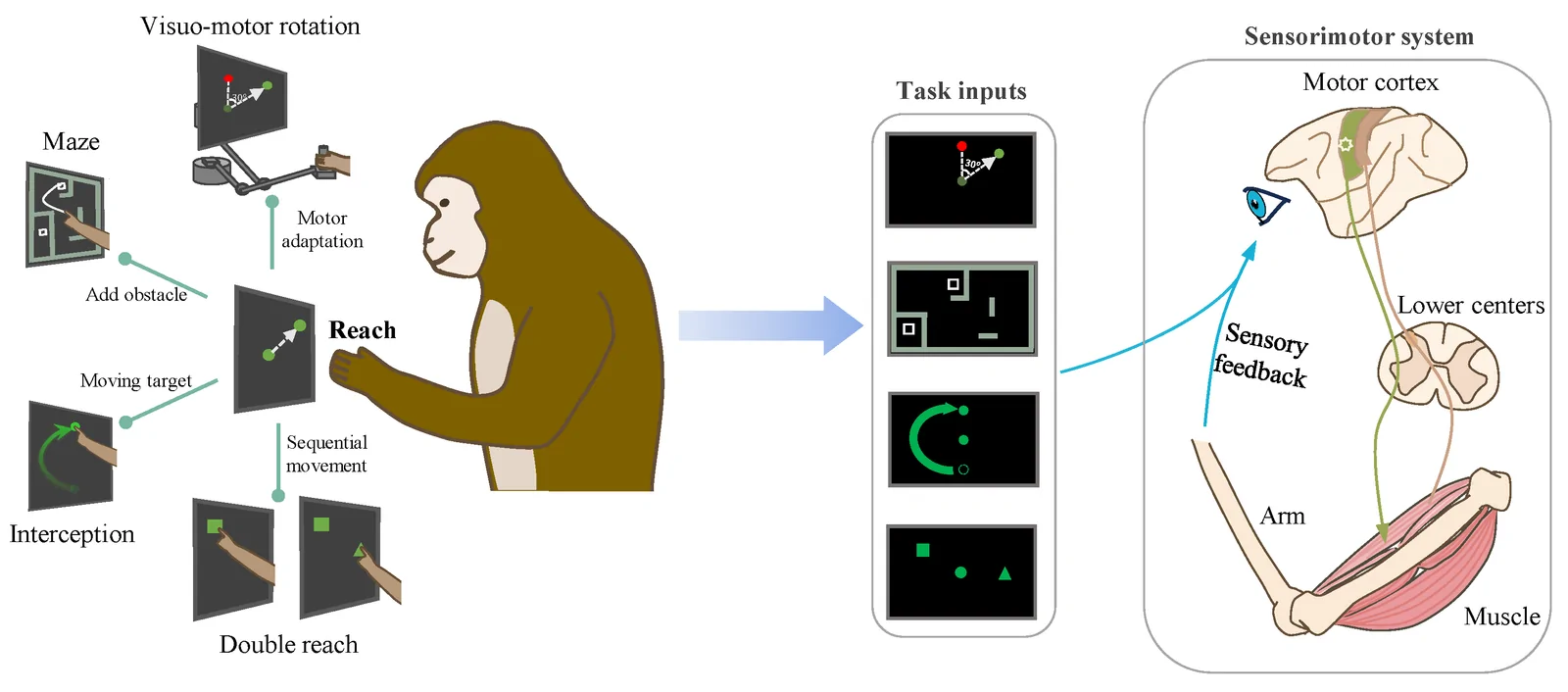
Regardless of the specific behavioral paradigm: visuo-motor rotation, obstacle avoidance, intercepting moving targets, or sequential actions—the execution invariably relies on reaching, a fundamental primitive natively embedded within the animal’s motor repertoire (**left**). Throughout the learning process, the subject primarily internalizes the high-level abstract rules governing the task. This paradigm was conceptualized schematically (**right**): the sensorimotor system is formulated as a closed-loop, dynamical control system endowed with foundational motor skills, which dynamically generates behavioral outputs under the joint modulation of sensory feedback and task-specific contextual inputs.

Parallel efforts in embodied motor control have shown that motor control cannot be understood in isolation from the biomechanical system being controlled. The development of differentiable musculoskeletal simulators—including MotorNet [36] and μSim [37]—has enabled end-to-end training of neural controllers that drive biomechanically realistic effectors. These frameworks have revealed that incorporating musculoskeletal dynamics qualitatively changes learned control solutions: networks controlling an arm exhibit different dynamical motifs than those controlling an eye [38], and proprioceptive feedback [39,40] or task inputs [41] shape neural representations in ways absent in purely kinematic models. Despite these advances, existing embodied modeling studies typically train their controllers from random initialization on a single target task, contrasting sharply with biological development wherein motor circuits are shaped by diverse sensorimotor experience prior to any specific experimental paradigm [42,43]. To investigate this question, we first consider what occurs when animals are trained on various motor tasks. Taking the macaque monkey as an example, regardless of the paradigm, successful task performance requires the monkey to possess a pre-existing motor capability: reaching, which has already been acquired. The entire training process can thus be viewed as the monkey embedding the task rule into its own sensorimotor system (broadly encompassing the cognitive system as well). Consequently, the activity patterns observed in the trained monkey’s motor cortex likely represent some form of fusion between the projection of task rules onto motor regions and of the intrinsic dynamics of the motor system itself (shown in Figure 1). A series of models was therefore constructed to simulate the above scenario as faithfully as possible and tested them on several classical tasks in the field of motor control.

Quantifying the similarity between model and biological neural representations requires metrics operating at two levels. At the level of neural geometry, canonical correlation analysis (CCA) on low-dimensional latent dynamics measures whether different systems visit similar neural states during behavior [44]. At the level of neural dynamics—asking not just which states are visited but how the system transitions between them—dynamical similarity analysis (DSA) provides a complementary framework by comparing Koopman operators after optimal alignment, yielding a distance metric that captures topological conjugacy [45]. Recent work has shown that dynamical similarity is dissociable from geometric similarity: two systems may occupy similar neural manifolds while evolving under fundamentally different rules [30]. This motivates the use of both metrics in evaluating model–brain alignment.

In the present work, we unify these threads by proposing a third paradigm—developmental pretraining—that differs fundamentally from both prior approaches. Unlike data-driven models, our model is never explicitly trained on neural data; instead, brain-like dynamics emerge as a consequence of acquiring a broad behavioral repertoire through embodied interaction. Unlike standard task-driven models that are randomly initialized and trained from scratch on a single constrained paradigm, our model first learns a diverse set of reaching skills across an entire workspace, simulating the developmental acquisition of motor primitives. The model was then evaluated systematically through CCA and DSA to determine the effect of pretraining within a closed-loop musculoskeletal control framework on alignment with primate motor cortical recordings across multiple task paradigms. Furthermore, neurophysiological evidence suggests that sequential reaching tasks are planned as integrated wholes rather than concatenations of independent segments [46,47], potentially relying on multiple timescale dynamics [48], metastable attractors [49], or thalamocortical loops [50]. To extend our investigation, whether single and double reach engage fundamentally distinct computational mechanisms was examined in the final part.

## 2. Materials and Methods

### 2.1. Model Architecture

**Figure 2:**
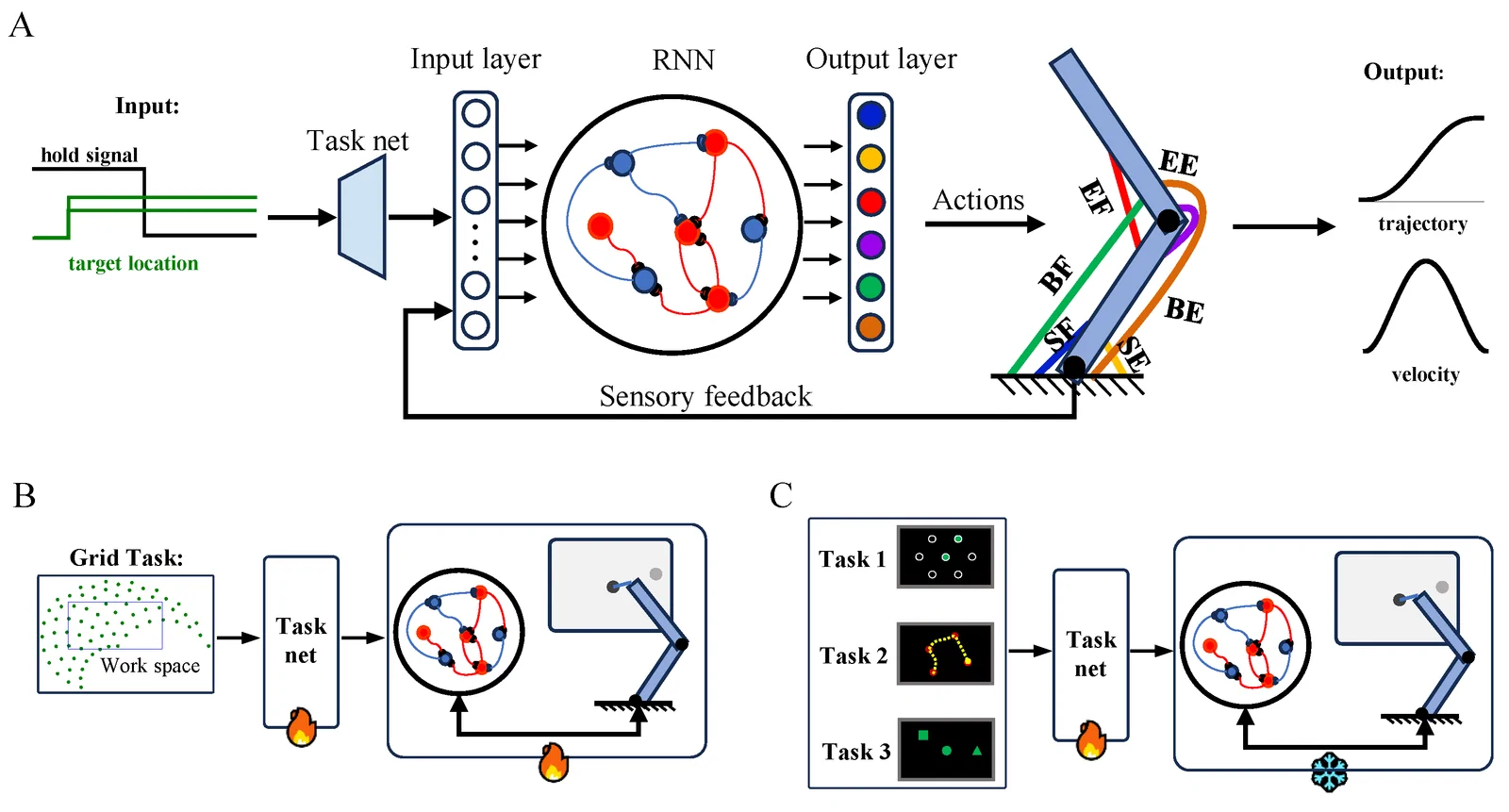
Model architecture and training pipeline. (**A**) The core model architecture comprises three interconnected modules: a recurrent neural network (RNN) controller, a task network interface (Task net), and a biomechanical musculoskeletal arm model. The task inputs, consisting of the hold signal and target location, are projected through the Task net to the RNN, which subsequently generates behavioral actions to drive the arm model, ultimately enabling the endpoint to execute the prescribed kinematic trajectories and velocity profiles. (**B**) Initially, all network parameters are optimized simultaneously via a grid-point reaching task to establish a foundational reaching model. (**C**) For downstream generalization tasks, all intrinsic recurrent weights within the RNN module are frozen, leaving only the parameters of the Task net open for training.

Inspired by the biological system mentioned in Figure 1, we constructed the following computational model (Figure 2A): we used an RNN to simulate the motor cortex, whose outputs drive a planar two-link musculoskeletal arm model to perform reaching tasks. Task-related inputs are processed by a dedicated task network and, together with visual and proprioceptive feedback, are fed into the RNN through an input layer. In the following sections, we will describe these components in detail.

#### 2.1.1. RNN Controller

To reflect key principles of cortical microcircuit organization while maintaining computational flexibility for complex task learning, the model implements an excitatory–inhibitory continuous-time recurrent neural network that incorporates several biologically plausible constraints [25]. The network consisted of N=100 recurrent neurons with an 80% excitatory ($N_{e}=0.8N$) and 20% inhibitory ($N_{i}=0.2N$) neuron ratio, according to Dale’s law. Each neuron was modeled as a hidden unit with fixed single-unit time constant τ=50 ms. The dynamics of the network’s N-dimensional hidden state vector h(t) were governed by

(1) $$\tau \frac{dh(t)}{dt}=-h\left(t\right)+f\left(W^{\mathrm{rec}}h\left(t\right)+W^{\mathrm{in}}u\left(t\right)+b+\sqrt{2\tau }\sigma_{\mathrm{rec}}\xi_{\mathrm{rec}}\left(t\right)\right),$$

where $W^{\mathrm{rec}}\in R^{N\times N}$ is the recurrent weight matrix, $W^{\mathrm{in}}\in R^{N\times M}$ is the input weight matrix (M=26 being the total input dimension), u(t) is the time-varying input vector, $b\in R^{N}$ is a bias term, and the activation function f(·) is tanh(·). The term $\xi_{\mathrm{rec}}\left(t\right)$ represents Gaussian noise with a standard deviation of $\sigma_{\mathrm{rec}}=0.15$ during training to promote robust learning. In practice the network needs to be discretized using the Euler method with a temporal discretization Δt=10 ms, yielding the update rule

(2) $$h_{t+1}=\left(1-\alpha \right)h_{t}+\alpha \cdot f\left(W^{\mathrm{rec}}h_{t}+W^{\mathrm{in}}u_{t}+b+\sqrt{2\tau }\sigma_{\mathrm{rec}}\xi_{\mathrm{rec}}\left(t\right)\right),\;\alpha =\frac{\Delta t}{\tau }.$$

The network output was a linear readout layer followed by a sigmoid nonlinearity σ(·):

(3) $$y_{t}=\sigma \left(W^{\mathrm{out}}h_{t}+b^{\mathrm{out}}\right).$$

where $W^{\mathrm{out}}\in R^{6\times N}$ is the output weight matrix, and $b^{\mathrm{out}}\in R^{6}$ is a constant bias.

#### 2.1.2. Musculoskeletal Arm Model

To better simulate the dynamics of brain-controlled arm movements, we employed the RNN controller to drive a musculoskeletal arm model. The PyTorch-based version of MotorNet (Version 0.2.0) [36] was utilized, a recently developed simulation toolbox for musculoskeletal systems, which provides a wide range of pre-implemented skeletal models, muscle models, and integrated arm assemblies. Specifically, we selected the RigidTendonArm26 effector, a planar two-joint arm actuated by six RigidTendonHillMuscle actuators: shoulder flexor (SF), shoulder extensor (SE), elbow flexor (EF), elbow extensor (EE), bi-articular flexor (BF), and bi-articular extensor (BE). This model offers a biomechanically realistic platform for simulating human upper-limb movements. All models in MotorNet are inherited from PyTorch’s nn.Module. Given muscle activations as input, the model automatically performs forward propagation to compute muscle and skeletal forward dynamics and kinematics, outputting movement parameters such as endpoint position and velocity, joint angles and angular velocities, muscle length, contraction velocity, and muscle force. Critically, the model is fully differentiable and participates in backpropagation during network training, allowing its outputs to be directly used in the loss function—greatly facilitating end-to-end learning. For ease of use, MotorNet also provides an Environment class that integrates the arm model with task specifications and sensory observation pipelines, enabling flexible selection of input/output parameters and the addition of customizable noise. In our setup, sensory feedback consisted of endpoint position (visual feedback) and muscle length and contraction velocity (proprioceptive feedback), combined with task-related inputs (described below) to form the observation vector. Outputs included endpoint position, velocity, and muscle forces. During training, both visual and proprioceptive feedback delays were set to 0 ms, with noise levels fixed at 0.10. During testing, noise was set to 0.

#### 2.1.3. Inputs to RNN and Task Network

The input vector u(t) comprised two main components, including sensory feedback and task instructions:

(4) $$u_{t}=\left[{\mathrm{fb}}_{t},\;\mathrm{task}\text{\_}{\mathrm{output}}_{t}\right].$$

As Section 2.1.2 mentioned, sensory feedback consisted of endpoint position (two dimensions) and muscle length and contraction velocity (6×2=12 dimensions). The task-specific component was generated by a dedicated feedforward network (tasknet) that processed abstract task instructions, $\mathrm{task}\text{\_}{\mathrm{input}}_{t}$, and consisted of target position, motor vigor and hold signal (four dimensions in most cases):

(5) $$\mathrm{task}\text{\_}{\mathrm{output}}_{t}=g_{\theta_{\mathrm{task}}}\left(\mathrm{task}\text{\_}{\mathrm{input}}_{t}\right),$$

where g(·) is a multi-layer perceptron with a tanh activation function. This architecture allows the RNN to receive high-level, processed (12 dimensions in most cases) rather than raw instructions. All network parameters are shown in Table 1.

#### 2.1.4. Parameter Initialization

All parameters were initialized as follows:

- **Recurrent weights**: The recurrent weight matrix $W_{\mathrm{rec}}$ follows a structured E/I pattern:
  $$W_{\mathrm{rec}}=\left[\begin{array}{cc} W_{EE} & -W_{EI}\\ W_{IE} & -W_{II} \end{array}\right]\in R^{N\times N}$$
  where all submatrices $W_{EE}\in R^{Ne\times Ne},W_{EI}\in R^{Ne\times Ni},W_{IE}\in R^{Ni\times Ne},W_{II}\in R^{Ni\times Ni}$ are initialized with non-negative elements.
  The weights are initialized with specific mean and variance to achieve a balanced network state:
  $$\begin{array}{ccc} \mu_{E} & =\frac{1}{\sqrt{N}},\; & \mathrm{Mean}\;\mathrm{for}\;\mathrm{excitatory}\;\mathrm{connections}\\ \mu_{I} & =\frac{Ne/Ni}{\sqrt{N}},\; & \mathrm{Mean}\;\mathrm{for}\;\mathrm{inhibitory}\;\mathrm{connections}\\ \sigma^{2} & =\frac{1}{N},\; & \mathrm{Variance}\;\mathrm{for}\;\mathrm{all}\;\mathrm{connections} \end{array}$$
  Each connection is sampled from a normal distribution, $w_{ij}∼N\left(\mu ,\sigma^{2}\right)$, with the appropriate μ depending on the connection type (E→E, E→I, I→E, I→I).
  To ensure dynamical stability, the recurrent weight matrix is normalized to have a specified spectral radius *r* = 1.5:
  $$W_{\mathrm{rec}}\leftarrow \frac{r}{\rho (W_{\mathrm{rec}})}\cdot W_{\mathrm{rec}}$$
  where $\rho \left(W_{\mathrm{rec}}\right)=\max_{i}\left|\lambda_{i}\left(W_{\mathrm{rec}}\right)\right|$ is the spectral radius of the weight matrix.
- **Input weights**: The input weight matrix $W_{\mathrm{in}}\in R^{N\times M}$ is initialized with excitatory connections only: $W_{\mathrm{in}}∼N\left(\mu_{E},\sigma^{2}\right)$.
- **Output weights**: The output weight matrix $W_{\mathrm{out}}\in R^{6\times N}$ connects only to excitatory neurons: $W_{\mathrm{out}}\left[:,1:N_{e}\right]∼N\left(\mu_{E},\sigma^{2}\right),\;W_{\mathrm{out}}\left[:,N_{e}+1:\right]=0$.
- **Bias**: The recurrent layer bias $b_{\mathrm{rec}}$ is initialized to 0 and the output layer bias $b_{\mathrm{out}}\in R^{P}$ is initialized to 0.6.
- **Initial state**: The network’s initial hidden state was drawn from a zero-mean Gaussian distribution with small variance: $h_{0}∼N\left(0,\sigma_{0}^{2}I\right),\;\sigma_{0}=0.1.$

### 2.2. Task Representations and Training Procedure

#### 2.2.1. Movement Trajectory and Velocity

To generate movement trajectories that more closely resemble those of human or animal subjects, we employed the minimum-jerk trajectory model. The minimum-jerk trajectory model proposed by Flash and Hogan [51] generates smooth reaching movements by minimizing the time integral of the squared jerk. Given an initial hand position $H_{1}=\left(x_{1},y_{1}\right)$ and a target position $H_{2}=\left(x_{2},y_{2}\right)$ with movement duration *t*, the trajectory is parameterized in normalized time:

(6) $$\tau =\frac{T}{t},\;T\in \left[0,t\right]$$

The hand position as a function of normalized time follows a fifth-order polynomial:

(7) $$H\left(\tau \right)=H_{1}+\left(H_{1}-H_{2}\right)\cdot \left(15\tau^{4}-6\tau^{5}-10\tau^{3}\right)$$

The velocity profile is obtained by differentiating the position equation with respect to actual time *T*:

(8) $$\dot{H}\left(T\right)=\left(H_{1}-H_{2}\right)\cdot \left(-\frac{30T^{4}}{t^{5}}+\frac{60T^{3}}{t^{4}}-\frac{30T^{2}}{t^{3}}\right)$$

#### 2.2.2. Grid Task

The core objective of this study is to endow a RNN with the capability to control a musculoskeletal arm model for forward dynamics. Specifically, given a target point location, the RNN should be able to guide the arm endpoint from any starting position to the target along a minimum-jerk trajectory. To achieve this, the network must learn a vast repertoire of point-to-point reaches. Our primary approach involves generating a set of grid points, using each point as a starting location and all other points as targets, thereby training the network to move between any pair within this grid.

- **Joint-Space Grid Points:** Points are generated based on the two joint angles of the dual-joint arm. The upper arm angle relative to the shoulder is $\theta_{1}$ ($0^{{}^{\circ}}<\theta_{1}<140^{{}^{\circ}}$), and the forearm angle at the elbow is $\theta_{2}$ ($0^{{}^{\circ}}<\theta_{2}<160^{{}^{\circ}}$). The endpoint in joint space is represented as $\left(\theta_{1},\theta_{2}\right)$. Values for $\theta_{1}$ and $\theta_{2}$ are sampled at fixed intervals Δθ, i.e., $\left(\theta_{1_{i}},\theta_{2_{j}}\right)=\left(i\cdot \Delta \theta ,j\cdot \Delta \theta \right)$. These joint angles are subsequently converted to the Cartesian coordinates of the arm endpoint. To avoid an impractically large number of points when Δθ is small, we constrain the selection to a defined Cartesian workspace (−0.3<x<0.3, 0.2<y<0.5 m, with the shoulder joint at (0,0)). Only points falling within this workspace are included in the training grid.
- **Cartesian Space Grid Points:** Similar to the joint-space method, but points are selected at fixed intervals directly within the Cartesian workspace. This method is commonly employed in non-human primate training experiments.
- **Random Grid Points:** Starting and target positions are randomly sampled within the Cartesian workspace.

For the latter two methods, since points are selected directly in Cartesian space, some locations may be kinematically unreachable by the arm model. These points require inverse kinematics to solve for the corresponding joint angles. Points yielding joint angles outside the feasible range are discarded. In this study, models were trained exclusively on the joint-space grid-point task. The Cartesian space and random point tasks were subsequently used to test whether the trained models acquired the generalized ability to move from any arbitrary starting point to any target within the workspace.

#### 2.2.3. Experimental Task

- **Center-out (CO) task:** The center-out paradigm is among the most prevalent experimental designs in motor control research. It is defined by a central starting point (x,y) and a set of targets uniformly distributed on a circle centered at this point with a radius *l* and an angular separation Δθ. Thus, the task is fully specified by these three parameters. In our implementation, Δθ was fixed at $45^{{}^{\circ}}$. Two variants were trained: a single-task model (CO Single) using one fixed parameter set, and a multi-task model (CO Multi) trained on multiple parameter sets (combining 4 different starting point locations and 3 target distances).
- **Random target touch (RTT) task:** A continuous random target task was employed. At the start of a trial, an initial starting point and a target appear at random locations within the workspace. Upon reaching a target, the next target immediately appears. All targets are confined to the workspace, with distances between consecutive targets ranging from 0.05 m to 0.15 m. The arm is required to initiate the next movement without delay, resulting in continuous motion throughout the trial.
- **Double-reach (DR) task:** The DR task is an extension of the CO task, featuring a fixed central starting point and two targets uniformly distributed around the circle. As a sequential task, it differs from the RTT: both targets are presented simultaneously at the trial’s onset. Following the “GO” cue, the model must execute two consecutive reaches—from the center to the first target, and then from the first target to the second—without receiving additional task-specific instructions. To accommodate the simultaneous input of two target coordinates, the input layer of the task network was expanded from 4 to 6 dimensions.

#### 2.2.4. Training Procedure

The RNN controller was trained to perform point-to-point reaching movements using a supervised learning paradigm. Training proceeded in two main phases: (1) an initial “growing up” phase to acquire basic motor control of single reach, which trained all parameters, both the RNN controller and the task network (Figure 2B), followed by (2) a fine-tuned training phase on specific behavioral tasks, which froze the RNN module and only updated the task network parameters (Figure 2C).

**Reaching Model Training:** Newly initialized RNN models were first trained on a generic reaching task. In each simulated trial, the model was required to move the arm endpoint from a random starting position to a random target position, both uniformly sampled from the joint space of the biomechanical arm model. To prevent the development of anticipatory movements, a catch trial paradigm was employed: in 50% of trials, no “GO” cue was provided. In valid trials, the “GO” cue was activated at a random time between 100 ms and 300 ms after trial onset.

**Loss Function:** The model was optimized using a composite loss function designed to shape realistic, efficient movements. The total loss *L* for a batch of trials was the mean over time steps *S* of a per-timestep loss $L_{t}$, which comprised several weighted components:

$$L=\frac{\sum_{t=1}^{S}L_{t}}{S},\;L_{t}=\lambda_{pos}L_{t}^{pos}+\lambda_{hold}L_{t}^{hold}+\lambda_{traj}L_{t}^{traj}+\lambda_{vel}L_{t}^{vel}+\lambda_{j}L_{t}^{j}+\lambda_{m}L_{t}^{m}$$

The primary components were:

- **Position Loss ($L_{t}^{pos}$)**: The $L_{1}$ norm between the actual endpoint position $x_{t}$ and the target position $x_{t}^{*}$. The target was the start position before the “GO” cue and the final goal position afterward.
- **Hold Loss ($L_{t}^{hold}$)**: The squared norm of the endpoint before the “GO” cue, penalizing movement during the preparation period.
- **Trajectory Loss ($L_{t}^{traj}$)**: The squared error against an ideal minimum-jerk trajectory during movement.
- **Velocity Loss ($L_{t}^{vel}$)**: The squared error against an ideal minimum-jerk velocity profile during movement.
- **Jerk Loss ($L_{t}^{j}$)**: The squared norm of the endpoint jerk (the third derivative of position), encouraging smooth, bell-shaped velocity profiles.
- **Muscle Activity Loss ($L_{t}^{m}$)**: Penalized the sum of squared muscle forces and their derivatives to promote low-energy, non-oscillatory muscle activations.

**Optimization Details:** Models were trained using the Adam optimizer with a learning rate specified in the training parameters on a NVIDIA RTX 5070Ti. Gradients were clipped to a maximum norm of 1.0 to ensure stable training. After each optimization step, specific architectural constraints (e.g., sparse connectivity) were enforced via a connectivity constraint method. Training typically consisted of 20,000 batches in the basic phase. Loss components were logged throughout training for monitoring convergence. The detailed parameters are listed in Table 2.

### 2.3. Quantification and Statistical Analysis

#### 2.3.1. Data Preprocessing

The neural data from the CO task was recorded in M1 and PMd using Utah array electrodes [52]. Five sessions from Monkey C were selected, with each session containing an average of 60.0±11.78 units and 118.4±16.86 trials, and 5 sessions from Monkey M, with each session containing an average of 40.8±8.52 units and 210.4±5.31 trials. The neural data from the RTT task was recorded in M1 and PMd using Utah array electrodes [53,54]. One session in M1 from Monkey M was chosen, comprising 61 units and 140 trials. The DR task data were obtained from an NKT dataset [46], comprising 118 units and 739 trials.

Raw spike counts were binned in non-overlapping 30 ms intervals (CO task) and 10 ms intervals (RTT and DR task) aligned to trial events. For each neuron, binned counts were first square-root transformed to stabilize variance. The time series was then smoothed by convolving it with a one-dimensional Gaussian kernel (standard deviation σ=50 ms). Neurons with an average firing rate below 1 Hz across the entire recording session were excluded from subsequent analysis.

To analyze condition-specific neural dynamics, we constructed trial-averaged firing rate matrices for defined epochs. For the execution-related analyses emphasized in this study, we extracted neural activity from a 1000 ms window beginning 500 ms before movement onset for the CO task and resampled the neural activity to 10 ms via cubic spline interpolation. For the DR task, we extracted neural activity from a 2300 ms window beginning 1300 ms before the first movement onset. Trial-averaged firing rates for each reach condition (e.g., each target direction) were computed within this window. For the RTT paradigm, the trial-averaged approach was inapplicable due to the inherent heterogeneity in trial composition, as each trial consisted of a variable number of reaches with distinct target locations and temporal durations. To standardize the data for analysis, we specifically isolated trials containing exactly four reaches. Subsequently, we aligned the neural activity for each trial using a fixed time window, defined by the shortest total duration observed across all selected trials.

Finally, a “soft” normalization was applied to the condition- and time-averaged firing rate matrix $F\in R^{n\times (c\cdot t)}$, where *n* is the number of neurons, *c* the number of conditions, and *t* the number of time bins per condition. The activity of each neuron was normalized by its firing rate range across all conditions and time points plus a constant offset of 5 Hz: $f_{\mathrm{norm}}=f/\left(\mathrm{range}\left(f\right)+5\right)$. The normalized rates were then mean-centered per neuron, resulting in a final data matrix X with zero mean for each neural dimension, ready for subsequent dimensionality reduction or similarity analysis.

#### 2.3.2. Neural Population Analyses

Dimensionality reduction techniques including principal component analysis (PCA) and jPCA [13] were used to visualize neural population activity. jPCA describes the temporal evolution of the neural state vector x(t) across time and conditions via a linear dynamical system: $\dot{x}\left(t\right)=Mx\left(t\right)$. The matrix M is constrained to be skew-symmetric to specifically capture oscillatory, rotational flow in state space. Prior to jPCA, the neural data (firing rates of *n* neurons) were projected onto their first 12 principal components to reduce dimensionality and focus on robust population-level signals. The analysis was performed on a 500 ms epoch spanning from 280 ms before to 220 ms after movement onset, a period of marked neural dynamics. Unlike the standard application of jPCA, the cross-condition mean activity was not subtracted. This allowed for direct comparison between the oscillatory modes identified by jPCA and the dynamics linearized around fixed points in our RNN models. The quality of the fit and the eigenvalues (which indicate oscillation frequencies) of the fitted M matrix were used as summary metrics.

#### 2.3.3. Canonical Correlation Analysis

To investigate the similarity of neural geometry across different models, a two-step dimensionality reduction and alignment procedure was implemented [44]. First, low-dimensional latent dynamics were estimated from the recorded neural populations of each individual session. For each session, PCA was applied first to *X* to define a low-dimensional neural manifold spanned by the leading *m* principal components (neural modes, where m=10). The high-dimensional neural activity was then projected onto these neural modes, yielding the latent dynamics matrix $L\in R^{m\times T}$. Next, canonical correlation analysis (CCA) was employed to linearly align the estimated latent dynamics from pairs of sessions across different individuals performing similar behaviors. CCA finds linear transformations that maximize the pairwise correlations (canonical correlations, CCs) between the two sets of latent signals. We use CCA to compute CC score on 10 PCs, then compute the Area Under Curve (AUC) of each CCA curve to quantify the similarity.

To quantify uncertainty, we performed a session-level bootstrap: for each condition, B=10,000 bootstrap samples were drawn by resampling the *N* sessions with replacement, recomputing the mean AUC per sample, and taking the 2.5th and 97.5th percentiles as the 95% confidence interval. For pairwise comparisons between a model and monkey data, the paired bootstrap difference $\Delta ={\mathrm{AUC}}_{\mathrm{model}}-{\mathrm{AUC}}_{\mathrm{monkey}}$ was computed over the same resampled sessions. The two-sided *p*-value was defined as twice the proportion of bootstrap differences with sign opposite to the observed mean difference [55].

#### 2.3.4. Dynamical Similarity Analysis

To rigorously quantify the similarity of internal computational processes at the level of temporal dynamics, dynamical similarity analysis (DSA) was implemented [45]. DSA operates by comparing the dynamical systems underlying the observed time series, rather than just their spatial geometries. The pipeline comprises two stages: (1) Extracting globally linear representations of the underlying nonlinear system via Koopman operator theory and Dynamic Mode Decomposition (DMD). Given a time series of neural states $X=[x_{1},x_{2},\ldots ,x_{T}]$, DMD finds the best-fit linear operator A that advances the state forward in time: $x_{t+1}\approx Ax_{t}$. This operator captures the essential dynamical characteristics of the system. (2) Comparing the resulting transition operators using an extension of statistical shape analysis customized for vector fields. The similarity between two dynamical systems is quantified by computing the Procrustes distance between their DMD operators after optimal alignment, yielding a scalar DSA score where lower values indicate greater dynamical similarity (i.e., the two systems are closer to being topologically conjugate).

(9) $$\mathrm{DSA}=\frac{∥{\tilde{K}}_{x}-Q{\tilde{K}}_{y}Q^{⊤}{∥}_{F}}{{∥{\tilde{K}}_{x}∥}_{F}+{∥{\tilde{K}}_{y}∥}_{F}},$$

where ${\tilde{K}}_{x}$ and ${\tilde{K}}_{y}$ are the reduced DMD operators and *Q* is the optimal orthogonal alignment matrix. Lower DSA scores indicate more similar dynamics.

Uncertainty was quantified via the same session-level bootstrap procedure (B=10,000, 95% percentile CI). Since different conditions sometimes had unequal numbers of sessions, unpaired bootstrapping (resampling each group independently) was used for DSA comparisons across conditions.

#### 2.3.5. Hypothesis Testing

The Friedman test (non-parametric repeated-measures analysis of variance) was used as the omnibus test for whether the *k* conditions differ in their central tendency across sessions. Under the null hypothesis that all conditions arise from the same distribution, the Friedman statistic follows a $\chi^{2}$ distribution with k−1 degrees of freedom; we additionally report the Iman–Davenport correction for improved small-sample behavior [56].

Post hoc pairwise comparisons were evaluated using bootstrap percentile methods as described above. All *p*-values from the five model-versus-monkey comparisons were corrected using the Holm–Bonferroni step-down procedure [57] to control the family-wise error rate at α=0.05.

For each model versus monkey comparison, we report the bootstrap mean difference Δ and its 95% confidence interval. The magnitude of deviation from the monkey baseline is directly interpretable from Δ (for CCA-AUC) or the raw DSA score (for DSA), both on their original measurement scales. All statistical analyses were performed using custom Python scripts (Python 3.11) built on NumPy [58], SciPy [59], and scikit-learn [60]. Bootstrapped confidence intervals and hypothesis tests are reported at the 95% confidence level unless otherwise stated. Multiple comparisons were controlled using the Holm–Bonferroni procedure [57].

## 3. Results

### 3.1. Behavioral Performance and Neural Activity of the Single-Reach Model

The reaching distance was first restricted within the workspace to a range of 0.05–0.35 m and constructed joint-space grid-point tasks across three distinct densities (designated as dense, moderate, and sparse). These tasks comprised 25, 15, and eight grid networks generated at joint angle intervals of 15°, 20°, and 25°, respectively, and were trained for 20,000 epochs. Through this optimization process, we aimed to endow the single-reach (SR) model with a foundational reaching capability; specifically, given the Cartesian coordinates of a target, the model should drive the endpoint of the musculoskeletal arm from any arbitrary initial position toward the target location, generating kinematics and trajectories that tightly adhere to the minimum-jerk profile.

**Figure 3:**
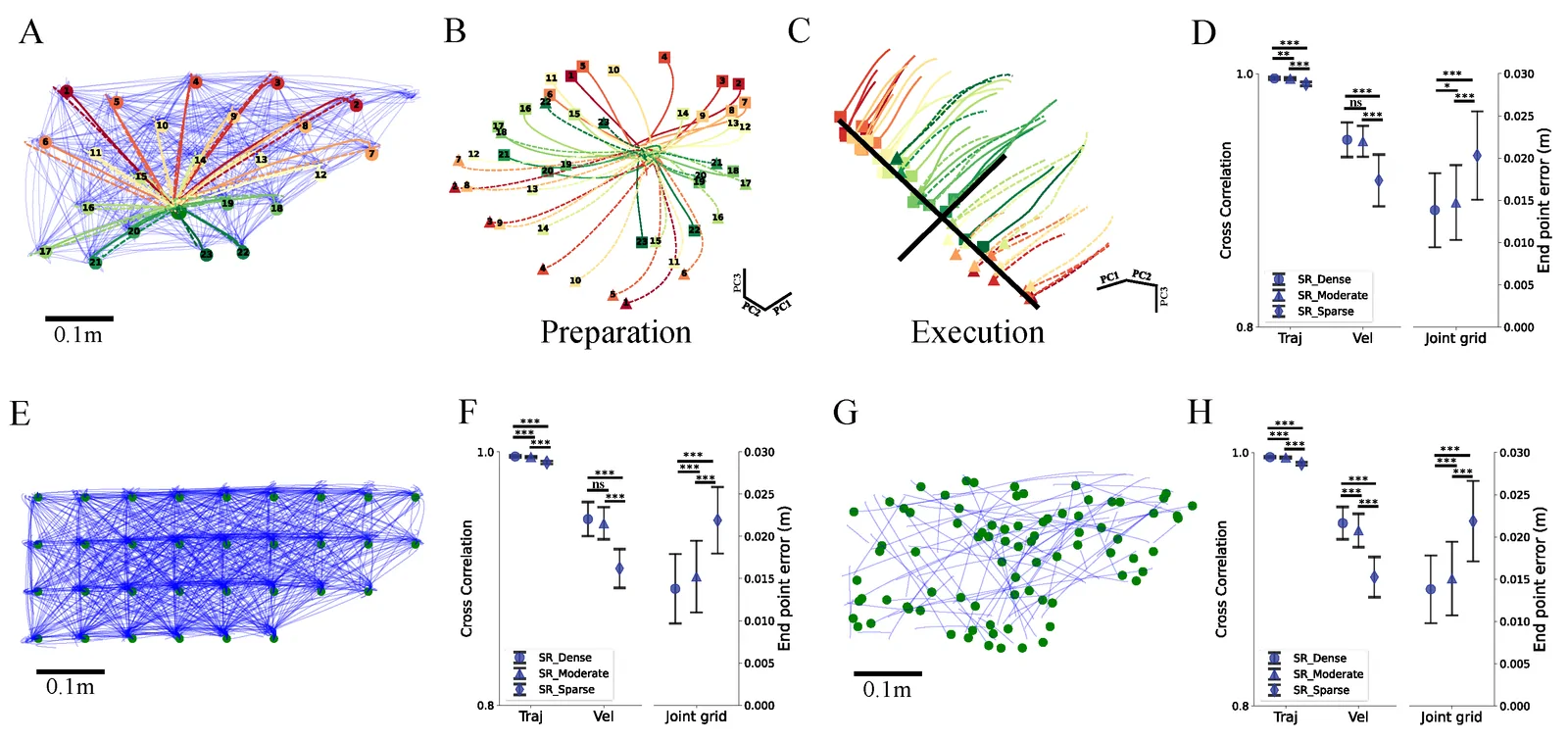
Behavioral performance and neural population dynamics of the reaching model. (**A**–**C**) illustrate a selected subset of target pairs within the joint-space grid task, displaying the endpoint kinematic trajectories, the neural trajectories during the motor preparatory phase, and the neural trajectories during the movement execution phase within a 3D principal component (PC) space, respectively. Numbers and colors designate specific target identifiers, while solid and dashed lines represent the forward and return trajectories for identical pairwise combinations. (**E**,**G**) depict the endpoint kinematic trajectories within the Cartesian space grid task and the random target grid task, respectively. (**D**–**H**) show the cross-correlation metrics for trajectories and velocity profiles, alongside the final endpoint errors, across the three task paradigms evaluated for all three model variants. *: p<0.05; **: p<0.01; ***: p<0.001; ns, not significant.

After training, we selected a focal subset of endpoints to examine the underlying population dynamics. We analyzed the RNN neural activity corresponding to all trajectories derived from pairwise permutations of starting and target locations within this subset (Figure 3A) utilizing principal component analysis (PCA). Notably, the top 10 principal components accounted for over 90 per cent of the total variance. For each reaching set (represented by solid or dashed lines), the distribution of neural states arrived at the end of the motor preparation phase preserved a topological geometry identical to that of the physical target locations (squares) or initial positions (triangles) (Figure 3B). Furthermore, neural trajectories corresponding to reciprocal movement pairs exhibited a centrally symmetric distribution within the neural state space, demonstrating that transposing the initial and target locations projects the initial motor state in the preparatory subspace along diametrically opposite directions. During the subsequent execution phase, the dynamical evolution of these neural trajectories proceeded along trajectories that were mutually orthogonal to the preparatory subspace (Figure 3C).

The behavioral performance of the models trained across the three grid densities on the training task is illustrated in Figure 3D. Models trained on the 25- and 15-point grid tasks both exhibited robust performance, yielding tracking and endpoint errors that were significantly lower than those observed in the eight-point grid task. To evaluate generalization, we subsequently tested the three SR models using Cartesian grid points with varying spatial intervals (Figure 3E) as well as randomly generated initial and target positions (Figure 3G). The models yielded robust performance consistent with their training baselines (Figure 3F,G). Collectively, these behavioral and neural population features indicate that the SR model has successfully acquired the capacity to generate biomechanically plausible trajectories across the entire workspace in a manner that closely recapitulates the dynamical signatures observed in the biological motor cortex.

### 3.2. The SR Model Shows Enhanced Alignment with Biological Neural Geometry and Dynamics

**Figure 4:**
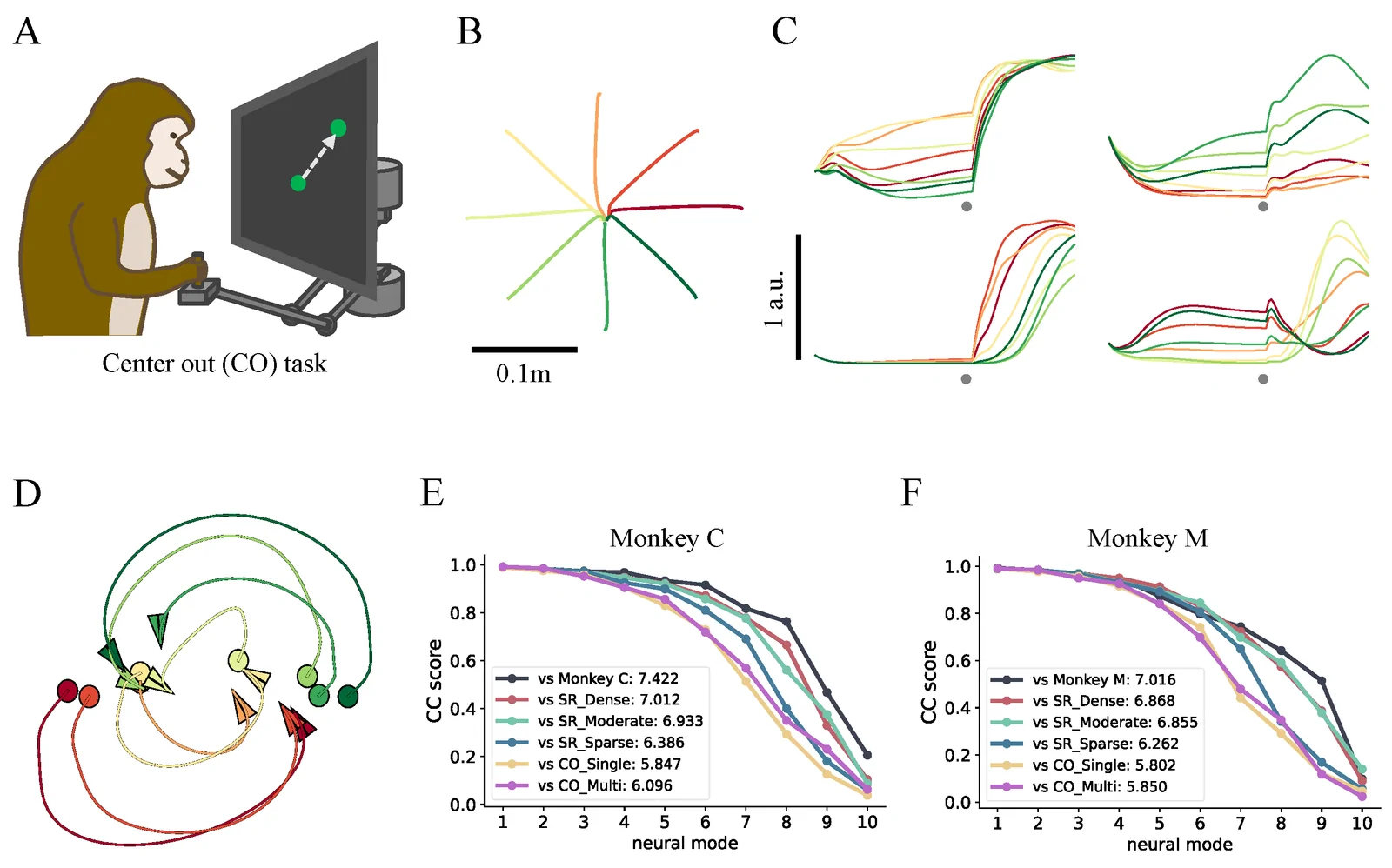
Model alignment with real data in the center-out (CO) task. (**A**) Schematic of the 8-direction CO paradigm. (**B**) Kinematic trajectories for a set of CO reaches. (**C**) Firing rate profiles of four representative recurrent nodes exhibiting distinct cortical tuning properties. Each profile corresponds to the reaching of the matching color in (**B**). (**D**) Population-level rotatory dynamics derived from applying jPCA to the neural activity during the execution phase. Each rotational trajectory corresponds to the reaching of the matching color in (**B**). (**E**) Canonical correlation analysis (CCA) metrics compared all candidate models against empirical cortical recordings from Monkey C. Specifically, the profiles illustrate the distribution of canonical correlation (CC) scores with the neural modes for each network variant, while quantifying these profiles by displaying the Area Under the Curve (AUC) for the respective CC score trajectories shown in the legend. (**F**) Same as (**E**) for Monkey M.

Following the validation of the SR models, we sought to benchmark their neural features against experimentally recorded cortical data during a canonical eight-direction center-out (CO) reaching task (Figure 4A). Because the CO task inherently simplifies to reaching movements directed from a central origin to radially distributed peripheral targets, the SR model successfully executed the paradigm zero-shot—completely bypassing the need for additional training or parameter optimization (Figure 4B).

During the CO task, the RNN exhibited highly heterogeneous single-unit activation profiles that closely recapitulated biological hallmarks of the motor cortex (Figure 4C). Specifically, Node 100 exhibited prominent tuning exclusively during the preparatory phase, which collapsed upon movement execution, whereas Node 32 remained quiescent during preparation but became strongly modulated during movement execution. Node 13 demonstrated consistent directional tuning across both epochs; conversely, while Node 4 was active throughout both periods, its preferred direction underwent a distinct tuning reversal following the “GO” cue. Furthermore, applying jPCA to the population activity during the execution phase unveiled the characteristic rotational dynamics typical of motor cortical populations (Figure 4D). Collectively, these findings qualitatively confirm the dynamical alignment between the SR model and its biological counterpart.

To establish a rigorous quantitative link, we interrogated whether the pretrained SR model serves as a superior computational proxy for the motor cortex compared to models trained exclusively on the CO paradigm. For a controlled comparison, we trained a model variant solely on the standard CO task (designated as CO Single). Additionally, by systematically varying task parameters—namely the central starting position and reaching distance—we generated a library of 12 distinct CO tasks to train a multi-task variant (designated as CO Multi). CCA (Method 2.3.3) was performed to align the neural state spaces of both Monkey M and Monkey C with the latent spaces of all candidate models. We then computed the distributions of canonical correlation (CC) scores across different recording sessions for the animal-to-animal and animal-to-model comparisons, quantifying these profiles via the Area Under the Curve (AUC).

**Figure 5:**
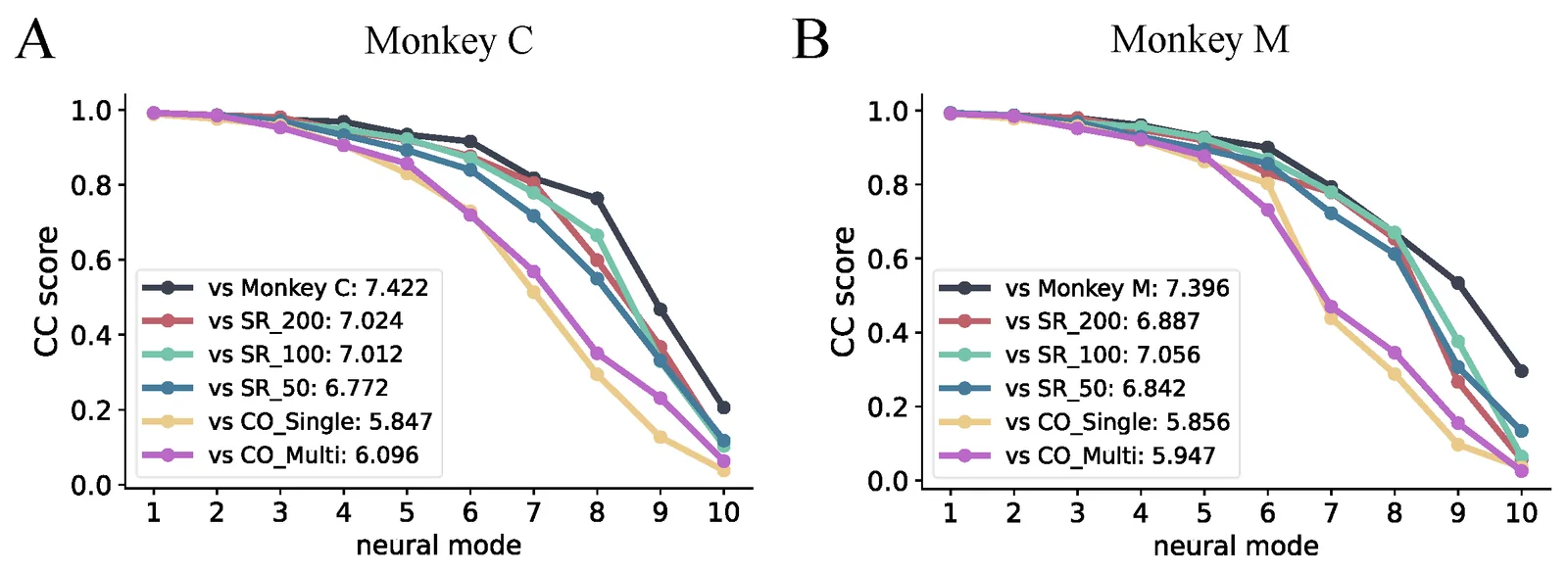
Different size models’ alignment with real data in the center-out (CO) task. (**A**) Canonical correlation analysis (CCA) metrics compared all candidate models against empirical cortical recordings from Monkey C as Figure 4E. (**B**) Same as (**A**) for Monkey M.

As illustrated in Figure 4E, all models show high consistency with the baseline (defined by cross-session comparisons of Monkey C) within the first three neural modes, while significant discrepancies emerge starting from the fourth mode. In terms of the AUC metrics, the SR models consistently outperform the CO models. Within each model group, models with more training data exhibit superior performance. A simple ablation experiment further shows that this result is independent of RNN size (shown in Figure 5). Across both monkeys, the CCA-AUC scores ranked the models in a consistent order (Friedman test, $\chi^{2}\;\;=\;\;36.6$, p<0.001 for Monkey C; $\chi^{2}\;\;=\;\;22.5$, p<0.001 for Monkey M). The SR models consistently achieved higher alignment than the CO variants (Table 3).

DSA scores–measuring the distance between linearized dynamical operators–produced a fully consistent ranking, with SR Dense yielding the closest match to monkey neural dynamics. All seven model types exhibited significantly different DSA profiles (Friedman test, $\chi^{2}\;=\;24.0$, p<0.001 for Monkey C; $\chi^{2}\;=\;23.7$, p<0.001 for Monkey M). Every pairwise model comparison was significant (bootstrap difference test, Holm-corrected p<0.001) for both monkeys, with the sole exception of CO Single versus DR Multi in Monkey M (p=0.516). The DSA further revealed that SR models form a distinct family that is significantly closer to neural dynamics than CO and DR models (Kruskal–Wallis test, H=19.9, p<0.001; mean DSA across families: SR 0.045±0.010, CO 0.076±0.014). Within the SR family, increasing sparsity monotonically degraded dynamical similarity (DSA: Dense 0.032 < Moderate 0.047 < Sparse 0.052), suggesting that denser recurrent connectivity better captures the rich temporal structure of cortical population dynamics.

Taken together, the CCA-AUC and DSA results converge on the same conclusion: the SR model–particularly its dense variant–achieves superior alignment with the geometric and dynamical properties of biological neural populations, and this superiority is statistically robust across two monkeys and multiple complementary metrics. These results indicate that even when performing only the CO task, the neural geometry and dynamics of the SR model was nearly identical to that of the real data, and was significantly better than that of models trained exclusively on the CO task. These CO models may have learned only the most basic task-relevant information, such as the first three dimensions of canonical correlation (CC), whereas the higher dimensional neural activity likely contains richer information. Moreover, taken together with the finding that the CO Multi model outperformed the CO Single model, this suggests that models trained on a single task paradigm primarily acquire information related to task rules, while the use of training data with a broader distribution enables the model to learn capabilities associated with specific motor control, thereby improving the similarity between the model and the real data.

### 3.3. The Fine-Tuned SR Model Exhibits Higher Dynamical Similarity in Random Target Tasks

**Figure 6:**
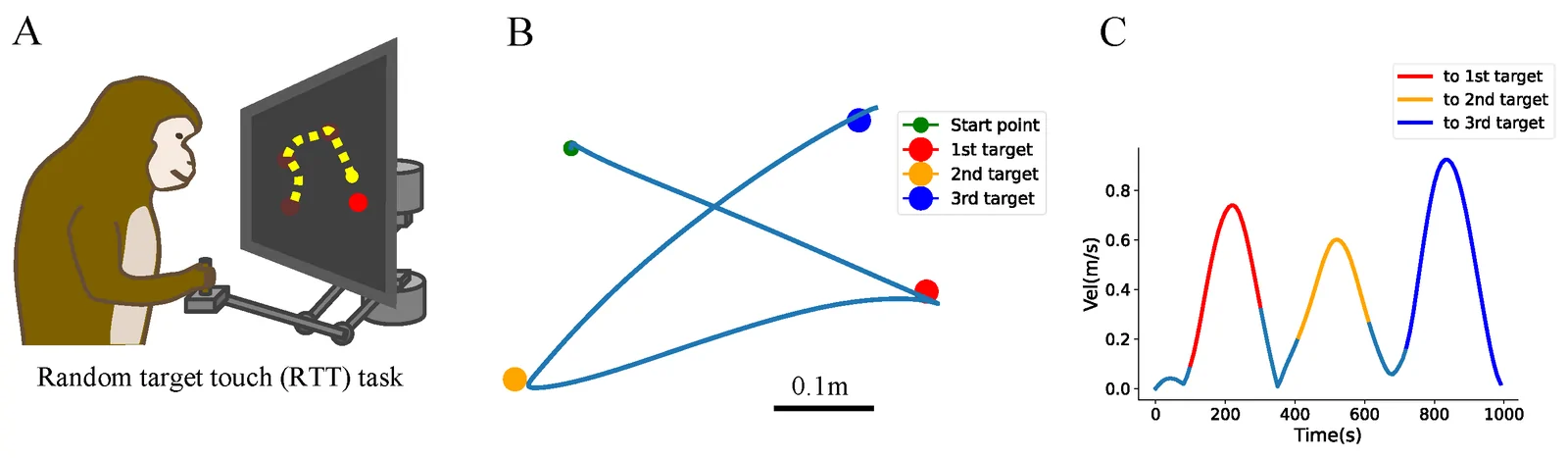
Behavioral performance within the random target task (RTT). (**A**) Schematic of the RTT paradigm. (**B**) Representative kinematic trajectory from a single, continuous RTT trial. (**C**) Velocity profile corresponding to the single-trial trajectory shown in (**B**).

The investigation was next extended to a more complex and dynamically demanding paradigm, the random target task (RTT), which explicitly evaluates the network’s capacity to generate continuous, sequential movements. In the RTT paradigm, the appearance of a novel target is triggered instantaneously upon the successful acquisition of the preceding one; consequently, the model must rapidly transition from the cessation of one reaching movement into the initiation of the next, thereby sustaining a fluid behavioral sequence (Figure 6A). To implement this, we structured each trial to consist of three sequential target presentations. Crucially, all intrinsic recurrent connection weights within the pretrained SR models were frozen, optimizing exclusively the parameters of the task network layer to map the sequential instructions onto the existing state space. For a rigorous baseline comparison, an RTT model was trained entirely from scratch utilizing an identical structural architecture.

Following training, all candidate networks successfully mastered the sequential paradigm; Figure 6B,C illustrate representative kinematic trajectories and velocity profiles generated by the fine-tuned SR Dense variant during a single trial. Given the inherent stochasticity of the RTT paradigm which was characterized by substantial trial-to-trial variance in targets and reach duration, the traditional trial-averaged alignment methods are not as effective as DSA. The RTT model trained from scratch exhibited a higher dynamical dissimilarity from the biological data compared to the fine-tuned SR models (shown in Table 4). These results support the reasonable inference that the SR model successfully instantiated a robust dynamical structure that closely recapitulates the fundamental computational mechanisms deployed by the biological motor cortex during continuous motor execution.

### 3.4. Sequential Movement Requires Different Underlying Computational Mechanisms

**Figure 7:**
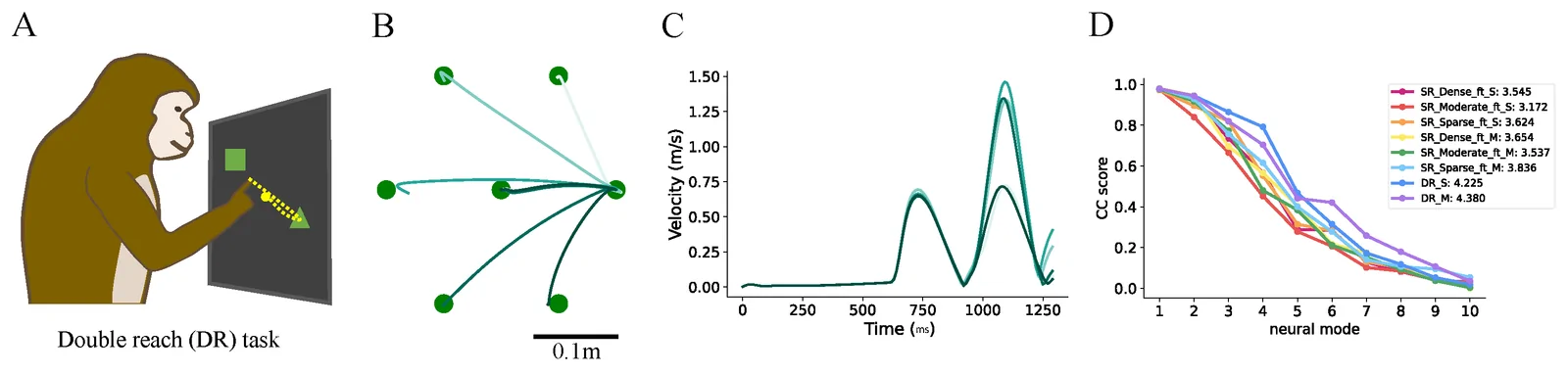
Model alignment within the double-reach (DR) task. (**A**) Schematic of the DR paradigm. (**B**) Kinematic trajectories for a set of DR tasks. Trajectories of different colors correspond to different second-movement targets. (**C**) Velocity profile corresponding to the single-trial trajectories shown in (**B**). (**D**) CCA curves and AUC for each network variant.

Lastly, we turned our attention to a more complex yet more commonly occurring behavior: sequential movement. Distinct from the previously examined continuous-reach task (RTT), sequential movement requires the subject to explicitly specify all subsequent motor targets prior to movement initiation and execute the entire sequence in a single, uninterrupted behavioral cascade, a feature that closely recapitulates the motor repertoires utilized in daily life. Here, we investigated the simplest instantiation of sequential movement: a double-reach (DR) task (Figure 7A). Similar to the CO paradigm, the spatial locations of two consecutive targets were presented simultaneously during the preparatory phase, rather than triggering the second target post hoc, requiring the network to execute two contiguous reaches during the execution phase. To accommodate the simultaneous input of dual-target coordinates, the input layer width of the task network was expanded to six while leaving all other parameters invariant. Mirroring the CO training protocol, we systematically manipulated the central starting position, reaching amplitude, and angular intervals of the peripheral targets to construct a library of 16 distinct DR task configurations. Each pretrained SR model was then fine-tuned under both single- and multi-task configurations. Together with two baseline DR models trained entirely from scratch, a total of eight model variants were evaluated.

Following training, all network cohorts successfully mastered the DR paradigm; Figure 7B illustrates representative kinematic trajectories directed toward identical initial targets, with corresponding velocity profiles depicted in Figure 7C. CCA and DSA were subsequently deployed across all models. Paradoxically, the results from the DR paradigm inverted the trends observed in both the CO and RTT tasks: the model trained from scratch exhibited a significantly higher degree of alignment with the empirical cortical recordings at both the neural geometry and neural population dynamics levels (Figure 7D). The same result also emerged in the DSA (SR Single: 0.0999, 0.0668, 0.0967; SR Multi: 0.0967, 0.0756, 0.0836; DR Single: 0.0493; DR Multi: 0.0246). This unexpected divergence implies that a single reach and a double reach may constitute two fundamentally distinct motor primitives. Consequently, the capacity to combine two independent movements into a cohesive compound sequence may rely inherently on the foundational, hardwired computational mechanisms embedded within the motor circuitry. Concurrently, the DR Multi model outperformed the DR Single variant, reinforcing our prior observations in the CO task that expanding the spatial distribution and diversity of training data drives the underlying network representations closer to biological plausibility.

To further validate the intrinsic dynamical discrepancies between the SR and DR models, we performed a reverse-transfer experiment by fine-tuning the from-scratch trained DR model on the standard CO task, reverting the task network input width back to four while freezing the intrinsic recurrent weights. Notably, the fine-tuned DR model exhibited the poorest alignment with both primates. Intriguingly, the DR Multi model, which previously demonstrated the highest similarity during the DR task, displayed the largest dynamical discrepancy from the empirical data when restricted to a single-reach behavior (DR Single: 0.076 for Monkey C, 0.081 for Monkey M; DR Multi: 0.088 for Monkey C, 0.0918 for Monkey M). This deficit demonstrates that the DR model instantiates a fundamentally different underlying computational mechanism compared to the SR model, providing compelling evidence that isolated movements and multi-component sequences are governed by segregated motor primitives.

## 4. Discussion

In this study, we developed a reaching model that mimics an animal’s movement system. By pretraining an RNN controller to control a biomechanical arm across a broad workspace—simulating an animal’s acquired motor repertoire—a form of “motor experience” that shapes its internal dynamics in ways that mirror biological development was embedded into the network. The controller can rapidly adapt to new task paradigms by fine-tuning only the task-input layer. Further results demonstrated that such “pretraining” leads to neural representations and dynamics that more closely resemble those observed in biological neural circuits. Finally, our results suggest that the ability to combine single reaches into sequential double reaches may rely on computational mechanisms inherent to the motor cortex itself.

### 4.1. Pretraining as a Computational Analog of Developmental Motor Learning

Recent years have seen a growing body of work in which motor primitives are acquired through training RNNs to improve generalization capacity [21,61,62,63,64]. However, these studies have typically relied on relatively simple tasks, such as eight-direction center-out reaching, and have required additional methods—for instance, initial state search or gain modulation—to generalize to other movement patterns. In the present work, we trained the model on a grid-point reaching task, with the goal of enabling the model to acquire fundamental reaching capability through training samples of larger scale and broader distribution. From a machine learning perspective, this step can be regarded as pretraining, a strategy that has been widely adopted in the field of artificial intelligence. The rapid advancement of pretraining methods in large-scale artificial neural networks—particularly in domains such as natural language processing and computer vision—has demonstrated that exposure to broad and diverse datasets allows models to acquire rich and generalizable representations. In large language models, for instance, unsupervised learning on massive unlabeled text corpora enables the extraction of statistical regularities and generative probabilities of linguistic sequences, providing high-quality parameter initialization that offers a superior starting point for subsequent fine-tuning in specialized domains, thereby accelerating convergence and conserving resources [65,66,67]. At the same time, a small number of studies have pointed out that these large models exhibit certain similarities with real brains at some levels [68,69]. Within computational neuroscience, however, pretraining has been employed in only a very limited number of studies [70]. Our work extends this concept to the modeling of motor neural control, demonstrating that pretraining an RNN on a broad motor skill repertoire of reaching movements markedly enhances the similarity between its internal dynamics and the neural activity recorded from the motor cortex.

Furthermore, across both the CO and DR models trained from scratch, the multi-task variants consistently outperformed their single-task counterparts. This phenomenon aligns fundamentally with the core computational logic of pretraining: by initially exposing the network to a broad parametric distribution of movements across diverse experimental configurations, the architecture is forced to internalize generalized motor representations and foundational dynamical manifolds prior to adapting to specific task constraints. In this context, our grid-point pretraining task represents a large-scale, systemic acquisition of generalized capacities. Symmetrically, multi-task training facilitates knowledge sharing and cross-task feature reuse within a localized behavioral family; thus, the underlying mechanics of knowledge recycling are conceptually harmonious. In other words, multi-task training can be fundamentally conceptualized as a form of weak pretraining. Devoid of an independent, serialized pretraining phase, it achieves inductive transfer concurrently alongside target task optimization. Consequently, its training intensity, behavioral coverage, and generalization ceiling remain inherently lower than those of a dedicated, ontogenetically inspired pretraining regime. Ultimately, multi-task learning operates as a lightweight, concurrent warm-up phase rather than a comprehensive developmental foundation.

When applied to RNNs used for simulating the brain, pretraining can be viewed as an in silico analog of developmental processes, simulating the years of sensorimotor experience that shape the connectivity and dynamics of biological neural circuits. In both the CO and the RTT tasks, the pretrained RNN exhibited neural geometry and dynamics that were more consistent with primate data than those of networks trained from scratch. This suggests that pretraining encourages the RNN to adopt computational strategies that have been optimized through evolution and development—strategies that may include efficient state-space organization, noise robustness, and the capacity to reuse existing dynamics for novel tasks. By starting from a pretrained network and proceeding to fine-tune it on specific tasks, researchers can more clearly dissect how task-specific fine-tuning modifies pre-existing dynamics, rather than studying networks whose dynamics have been dominantly shaped by a single task. This approach aligns with recent directions in neuroscience concerning “meta-learning” [71] and “foundation models” [72], in which adapting a common network to diverse experimental conditions enables clean cross-task comparisons and the effective isolation of task-invariant neural mechanisms.

### 4.2. Sequential Movements Are Fundamentally Distinct from Single Movements

It is noteworthy that in the DR task, despite possessing a rich repertoire of single-reach primitives, the pretrained model performed markedly worse in matching neural dynamics compared with the DR model trained from scratch, which stands in stark contrast to the preceding results. One interpretation is that sequential movements are not merely concatenations of individual motor commands mediated by external task inputs, but rather require a distinct form of internal dynamics that integrates multiple spatial goals into a coherent motor plan prior to execution. This view is supported by neurophysiological evidence showing that motor cortex neurons can encode an entire sequence as a unified representation, rather than as independent segments. This interpretation is consistent with the theoretical framework of dynamic primitives, which posits that discrete point-to-point movements and sequential compound actions belong to fundamentally distinct primitive classes with different control policies [73]. Neurophysiological evidence from human fMRI studies further supports this dissociation: primary motor cortex (M1) activity patterns for multi-movement sequences can be fully explained by a linear combination of patterns for the constituent single movements, whereas premotor and parietal areas encode genuine sequence-level information beyond elemental contributions [74,75]. Moreover, the representational content in these regions undergoes a dynamic state shift from independent feature encoding during planning to integrated encoding during execution—a process unique to sequential movements and absent in discrete single reaches [76]. Non-human primate studies have demonstrated that preparatory activity in M1 and PMd can reflect the structure of the entire sequence, that upcoming movements influence neuronal encoding, and that encoding patterns undergo shifts during movement execution [46]. At the neural population level, the preparation for the second movement occurs during the execution phase of the first [47]. This capacity for sequence integration and concurrent preparation during ongoing movement may depend on specific circuit architectures or dynamical motifs. In several network modeling studies, multiple-timescale neural network models [48], metastable attractor structures [49], and thalamocortical loops [50] have all been shown to contribute to the generation of sequential movements. Furthermore, the fine-tuned DR model also underperformed relative to the SR model when tested on the CO task, suggesting that future research should explore structured pretraining—that is, through task selection or curriculum design—to better capture the hierarchical and compositional nature of natural motor skill acquisition. This perspective holds important implications for both neuroscience and neural engineering. If sequence generation is indeed a core competency of the motor cortex, then brain–machine interfaces designed for sequential control may benefit from decoders that leverage these intrinsic dynamics, rather than relying solely on external cue-driven inputs. Likewise, neurorehabilitation approaches could aim to enhance or restore endogenous sequencing mechanisms within motor circuits.

### 4.3. RNN Offers Advantages in Modeling the Motor Cortex and Controlling a Musculoskeletal Arm

In this study, we required the RNN to control a musculoskeletal arm model rather than directly outputting motion trajectories, velocities, or electromyographic signals. In recent years, an increasing number of studies have adopted arm models to simulate more realistic conditions as closely as possible [37,39]. Controlling an effector with inherent constraints and dynamic characteristics is more complex than directly generating motion parameters, resulting in significantly different network activity. Previous network modeling experiments have demonstrated that even controlling effectors with distinct characteristics, such as an arm versus an eye, leads to markedly divergent network dynamics [38]. From the perspective of embodied intelligence, intelligence does not exist in isolation within the brain but emerges from interactions between the body and the environment—particularly for motor intelligence [77], where physical manipulation is crucial.

According to optimal feedback control (OFC) theory, internal models can predict the sensory consequences of self-motion and integrate delayed, noisy sensory feedback (e.g., visual, proprioceptive) to form more accurate and timely estimates of the current limb state [78]. To investigate closed-loop sensorimotor control, a musculoskeletal system introduces realistic temporal delays, noise, nonlinearities, and redundancy—all critical factors shaping neural control strategies. Training an RNN to control such a system facilitates the emergence of robust and generalizable controllers with properties akin to the adaptive and fault-tolerant nature of biological motor control.

The integration of RNNs with musculoskeletal arm models provides a powerful framework for simulating how the motor cortex integrates innate dynamics with task-specific learning. This approach not only enhances model performance and neural fidelity but also offers a platform for generating testable hypotheses regarding the neural basis of motor adaptation, skill composition mechanisms, and the role of feedback in shaping cortical dynamics. Future research should expand this paradigm to more complex tasks, richer sensory inputs, and alternative learning frameworks such as reinforcement learning, thereby further bridging the gap between artificial and biological motor intelligence and providing models and theoretical architectures for the field of biomimetic robotic control.

### 4.4. Limitations and Future Work

This study presents several limitations that should be addressed in future work.

**Musculoskeletal modeling.** Our model employs a planar two-joint arm actuated by six muscles (RigidTendonArm26). While this provides a biomechanically grounded platform for closed-loop sensorimotor control, it is a substantial simplification of the human upper limb, which contains over thirty muscles with complex architecture including pennation angles, fiber-type heterogeneity, and multi-articular pathways. Prior work has shown that increasing the level of biomechanical detail can influence the neural control strategies learned by models [42,79], and the extent to which additional muscular detail would alter our conclusions about neural similarity and motor primitives remains to be determined. Incorporating more complete musculoskeletal models with additional degrees of freedom, such as wrist and hand effectors, would test the generality of our findings and enable investigation of dexterous manipulation.

**Sensory feedback delays.** We set visual and proprioceptive feedback delays to zero during both training and evaluation. In biological motor control, sensory feedback is delayed by approximately 50–150 ms for vision and 30–50 ms for proprioception. A large body of work has established that the brain compensates for these delays through internal forward models, likely implemented in the cerebro-cerebellar circuit [80,81]. Effective delay compensation requires at minimum a memory buffer of efference copies, a state observer for state estimation, and a predictor for forward simulation [81]. Patients with cerebellar damage exhibit increased phase lag in feedback control, consistent with loss of this predictive mechanism [82]. Models trained without delays may learn qualitatively different control strategies—reactive rather than predictive—potentially affecting their neural alignment with delay-aware biological circuits. Incorporating realistic delays in future work would test whether our model spontaneously develops forward-model-like computations and whether this further improves similarity with neural data.

**Scalability to complex behaviors.** Our model was evaluated on reaching and two-reach sequence tasks, which represent a narrow slice of the motor repertoire. Scaling to more complex, hierarchically structured behaviors—such as multi-step tool use, whole-body coordination, or interaction with movable objects—is unlikely to be achievable with a single-timescale RNN in a purely supervised learning paradigm. Prior work suggests that dedicated mechanisms for motif transitions [50] and multiple timescales of neural dynamics [48] are necessary for robust sequencing and hierarchical control. Modular architectures that separate encoding, scheduling, and motor execution have demonstrated compositional generalization from trained two-segment movements to untrained multi-segment sequences [83]. Future work should explore whether incorporating such architectural features into our pretraining framework enables scalable acquisition of complex behavioral repertoires.

**Neural datasets.** Our neural comparisons are based on a limited set of recordings: 10 sessions from two monkeys for the center-out task, one session for the random target touch task, and one dataset for the double-reach task (118 units). While manifold-level analyses such as CCA and dynamical similarity analysis are reasonably robust to cross-session and cross-subject variability [44,84], additional recordings across more subjects, tasks, and recording modalities would provide stronger validation of the model’s neural alignment and reveal potential failure modes. In particular, simultaneous recording from multiple cortical areas (M1, PMd, SMA, parietal cortex) during task performance would enable richer comparisons with the distributed representations in our model.

**RNN dynamics and network structure analysis.** Our current analysis approaches the pretrained RNN primarily as a black box, employing CCA and DSA to compare population-level latent dynamics with neural data, without attempting a systematic reverse-engineering of its internal computational mechanisms. A mature toolkit exists for opening this black box—fixed-point analysis to identify attractor structures and slow manifolds [29], Jacobian linearization to extract the input–output mapping around fixed points, and low-dimensional projection methods such as demixed PCA [85] to separate task–parameter representations. Applying these tools to our model could reveal whether pretraining induces dynamical motifs—such as rotational dynamics [20] or low-rank connectivity structures [86]—that mirror those observed in the motor cortex, and how these differ from the scratch-trained counterpart. Prior work has shown that independently trained RNNs converge to universal dynamical motifs on the same task [87], and that learning in the motor cortex involves reassociation of existing patterns rather than generation of entirely new neural activity [88]. Analyzing whether our pretrained model’s dynamics align with these principles would substantially strengthen the biological interpretability of the framework. Future work should also examine how the pretrained network flexibly combines dynamical subsystems across tasks [30], and whether its connectivity structure exhibits the low-rank separability that is thought to underlie neural computation in biological circuits [86,89].

**Biologically constrained RNNs.** A stronger form of biological constraint—connectome-constrained modeling—uses empirically measured anatomical wiring diagrams to determine the presence (or strength) of individual synapses. Pioneered in the Drosophila visual system, where a complete synapse-resolution connectome is available, this approach has shown that networks constrained by the optic lobe connectome can accurately predict neural responses across dozens of experimental conditions using only a handful of free parameters [90]. Theoretical work has established that connectome constraints alone are often insufficient to uniquely determine dynamics—a fixed wiring diagram admits many dynamical solutions—but recordings from a small subset of neurons can break this degeneracy [91]. In the mammalian brain, the MICrONS mouse visual cortex dataset—combining nearly 12,000 co-registered excitatory neurons with spatial coordinates, anatomical connectivity, and functional activity—has enabled biologically grounded RNNs whose weight initialization and spatial embedding reflect measured cortical wiring; these networks develop low-entropy, modular, small-world organization and outperform unconstrained baselines on cognitive decision-making tasks [92]. Incorporating connectome constraints into our pretraining framework—even partial ones, such as spatial embedding or motif statistics—would enable direct comparison of the learned weight matrix with anatomical connectivity data from the motor cortex, bridging task-performing RNNs and structurally grounded cortical microcircuit models.

**Brain-machine interfaces with dynamics-informed decoders.** Recurrent neural networks have become a popular architecture for closed-loop intracortical BMI decoding, consistently outperforming linear and transformer-based alternatives in online finger and cursor control tasks [93,94]. Our pretrained RNN—whose internal dynamics already mirror biological motor cortical activity—could serve as a structured neural prior for BMI decoders, reducing calibration time and improving cross-session generalization. This complements recent foundation model approaches: NDT3, a Transformer pretrained on over 2000 h of neural spiking data from 30+ monkeys and humans, has shown that large-scale pretraining benefits downstream decoding under real-world distribution shifts [95]. Similarly, POSSM combines spike tokenization with recurrent state-space models to achieve cross-species transfer of neural dynamics—from the monkey motor cortex to human handwriting decoders [96]. Our framework offers a complementary, hypothesis-driven path: pretraining in a closed-loop sensorimotor context may produce representations that are inherently more transferable to biological decoding, because they reflect the same computational constraints that shape real neural dynamics. Beyond offline decoding, online RNN frameworks such as RONDO improve decoding accuracy by 35–45% over offline methods within real-time constraints [97], suggesting that our continuously running RNN could be adapted for adaptive, closed-loop BCI operation. RNN decoders have also been successfully deployed for bimanual movement control from intracortical signals in human participants [98] and for high-performance continuous finger decoding enabling real-time quadcopter game control [99], demonstrating the maturity of this approach for clinical BCI applications.

## 5. Conclusions

In this work, we propose a promising model architecture that integrates pretraining with embodied RNN models, opening multiple new research avenues. These include extending pretraining to richer multisensory experiences, combining supervised pretraining with reinforcement learning, and utilizing pretrained networks as in silico testbeds for brain–machine interface algorithms. By grounding RNNs in realistic sensorimotor experience, we advance toward building models that not only perform tasks but also explain how neural circuits solve problems—a crucial step toward developing computationally principled theories of brain function.

## Figures and Tables

**Table 1:** Overall network architecture with layer dimensions.

Module	Layer	Input Dim	Output Dim
**Task Network**	Linear + Tanh	4 (or 6) ^a^	64
	Linear	64	12
**Input Layer**	Sensory feedback (FB)	14 ^b^	–
	Task output (concat.)	12	–
	Linear (no bias)	26	100
**Recurrent Layer**	Linear (no bias)	100	100
	Activation	tanh	
**Output Layer**	Linear + bias	*N*	6
	Activation	Sigmoid	

^a^ 4 for Basic/CO/RTT (1 target: 2-dim, “GO” cue: 2-dim); 6 for DR (two targets: 4-dim + “GO” cue: 2-dim). ^b^ 12 proprioception (6 muscle lengths + 6 velocities) + 2 vision (fingertip *x*, *y*).

**Table 2:** Training configuration.

Config	Basic (SR)	CO	RTT	DR
**Optimization**				
Optimizer	Adam	Adam	Adam	Adam
Learning rate	$1\times 10^{-4}$	$1\times 10^{-4}$	$1\times 10^{-4}$	$1\times 10^{-4}$
Gradient clip	1.0	1.0	1.0	1.0
Batches	20,000	20,000	5000	10,000
**Frozen params**				
Recurrent weight	— ^a^	Frozen	Frozen	Frozen
Output weight	—	Frozen	Frozen	Frozen
Output bias	—	Frozen	Frozen	Frozen
TaskNet	—	Trainable	Trainable	Trainable ^b^
**Loss weights**				
Position	$1\times 10^{2}$	$1\times 10^{2}$	$1\times 10^{2}$	$1\times 10^{2}$
Hold	$5\times 10^{2}$	$5\times 10^{2}$	—	$3\times 10^{2}$
Trajectory	$1\times 10^{3}$	$1\times 10^{3}$	—	$1\times 10^{3}$
Velocity	$5\times 10^{2}$	$5\times 10^{2}$	$1\times 10^{2}$	$5\times 10^{2}$
Jerk	$1\times 10^{3}$	$1\times 10^{3}$	$1\times 10^{4}$	$1\times 10^{1}$
Muscle	$1\times 10^{-2}$	$1\times 10^{-2}$	$1\times 10^{-2}$	$1\times 10^{-2}$
**Task config**				
Batch size	— ^c^	80/960	128	30
Task type	Grid reach	Center out	RTT	Double reach
Catch trial	0.1	0.1	0.0	0.0
Delay (s)	[0.3, 0.6]	0.6	0.0	0.6

^a^: All params trained (full training). ^b^: DR TaskNet has 6-dim input; all its params are trainable. ^c^: All trials in the task.

**Table 3:** CCA-AUC comparison between each model and monkey neural data. Δ denotes the mean paired difference from the monkey condition; *p*-values are Holm-corrected for five comparisons.

Model	Monkey C		Monkey M	
	AUC [95% CI]	Δ (p)	AUC [95% CI]	Δ (p)
Monkey	7.40[7.10,7.75]	—	7.27[7.01,7.50]	—
SR Dense	7.54[7.32,7.73]	+0.14 (0.362)	7.15[6.84,7.43]	−0.12 (0.053)
SR Moderate	7.46[7.24,7.65]	+0.06 (0.655)	7.13[6.76,7.42]	−0.14 (0.012)
SR Sparse	7.06[6.88,7.22]	−0.34 (0.021)	6.64[6.44,6.84]	−0.63 (<0.001)
CO Multi	6.92[6.72,7.12]	−0.47 (<0.001)	6.46[6.15,6.77]	−0.81 (<0.001)
CO Single	6.24[6.02,6.47]	−1.15 (<0.001)	5.85[5.68,6.03]	−1.42 (<0.001)

**Table 4:** DSA scores for RTT model variants (mean ± bootstrap 95% CI over N=10 seeds). Lower scores indicate greater dynamical similarity to monkey M1 data. All pairwise differences are significant (***: Holm-corrected bootstrap p<0.001).

Model	Mean DSA	95% CI	Pairwise Δ vs. Best
RTT_SR Dense	0.055	[0.054,0.056]	—
RTT_SR Moderate	0.060	[0.059,0.061]	+0.005 ***
RTT_SR Sparse	0.071	[0.070,0.072]	+0.016 ***
RTT_learn from scratch	0.101	[0.101,0.102]	+0.047 ***

## Data Availability

The original contributions presented in this study are included in the article. The custom code used for this study will be made publicly available on GitHub (https://github.com/RevuBu/Pretrained_RNN (accessed on 31 July 2026)) upon publication.
